# The overlap between binge eating disorder and substance use disorders: Diagnosis and neurobiology

**DOI:** 10.1556/JBA.2.2013.015

**Published:** 2013-10-15

**Authors:** Liana R. N. Schreiber, Brian L. Odlaug, Jon E. Grant

**Affiliations:** ^1^Department of Psychiatry, Impulse Control Disorder Clinic, University of Minnesota, Minneapolis, MN, USA; ^2^Department of Public Health, Faculty of Health & Medical Sciences, University of Copenhagen, Copenhagen, Denmark; ^3^Department of Psychiatry & Behavioral Neuroscience, University of Chicago, Chicago, IL, USA

**Keywords:** addiction, binge eating disorder, comorbidity, substance use disorder

## Abstract

*Background and aims:* Binge eating disorder (BED) is a relatively common condition, especially in young adult females, and is characterized by chronic over-consumption of food resulting in embarrassment, distress, and potential health problems. It is formally included as a disorder in DSM-5 for the first time, an acknowledgement to its debilitating nature. This article explores the overlap between binge eating disorder and substance use disorders (SUD). *Methods:* The bibliographic search was a computerized screen of PubMed databases from January 1990 to the present. Binge eating disorder, substance use disorder, binging, obesity, food addiction, comorbidity, dopamine, opioid, serotonin, glutamate, and pharmacological treatment were the keywords used in searching. *Results:* BED shares similar phenomenology to SUD, including significant urges to engage in binging episodes, resulting in distress and impairment. Similar neurobiological pathways are found in both BED and SUD and medications based on similar neurobiology have been examined for both disorders. A subset of individuals with BED may have a “food addiction”, but there is no clinical agreement on the meaning of “food addiction”. Exploring the relationship between BED and obesity may also shed light on the extent to which BED can be viewed as an addiction. *Conclusions:* Overall, nascent research regarding BED and SUD suggests an overlap between these disorders, but there are discrepancies between these two disorders that need further exploration.

## INTRODUCTION

Binge eating disorder (BED) is a disorder characterized by the compulsive engagement in excessive food consumption which results in feelings of guilt or shame and, in many cases, physical problems (American Psychiatric Association, 2013). Despite the mirroring characteristics, such as increased tolerance to the amount of food or substances consumed, triggering urges to consume food or substances, and similar dopaminergic and opiate pathways for highly palatable foods and substances, between binge eating and substance addictions, exploring the intersect between these two is relatively recent. This article provides a brief overview of the current literature on BED and discusses the overlap of BED with substance use addictions (e.g., illicit drug, nicotine, and alcohol dependence; SUD) in order to better understand the nature of BED.

## METHODS

All literature was found through the search engine PubMed from January 1990 to the present. Keywords for locating articles included: binge eating disorder, binging, substance use disorder, obesity, food addiction, comorbidity, dopa-mine, opioid, serotonin, glutamate, and pharmacological treatment. The keywords “binge eating disorder”, “binging”, and/or “substance use disorder” were used alone in all searches and were combined with each of the previously mentioned keywords. Each search resulted in a varying number of articles with binge eating disorder and glutamate providing only 5 results, and substance abuse and pharmacological treatment resulting in 88,251 articles. Binge eating disorder and substance use disorder resulted in 151 articles. Articles reviewed for this publication were based on publication date, with articles from the year 2000 onwards as the focus, and the degree to which the article related to both binge eating disorder and substance use disorders. A total of 70 publications were reviewed for this paper, including 11 articles published between 1990 and 2000 that were addded due to the additional support they provided.

## EPIDEMIOLOGY AND PHENOMENOLOGY

BED occurs in about 1.4% (0.8% to 1.9%) of the global population and has a mean age of onset of approximately 23 years (Kessler et al., 2013). BED occurs more frequently in young adults (ages 18–29), females, and college-educated and student populations (Kessler et al., 2013). Interestingly, males and females may have similar binge episode frequency, but males report less distress compared to females, and therefore, do not meet full BED criteria (Lewinsohn, Seeley, Moerk & Striegel-Moore, 2002). Similar to BED, SUD peaks in late adolescence and young adulthood. Males with BED, however, are about two times more likely to have a SUD compared to females; yet, higher rates of specific addictions, such as cocaine, psychotherapeutic drugs, and persistent alcohol use disorder have been noted in females (Merikangas & McClair, 2012). People rarely seek treatment for binge eating or admit to it on direct questioning (Dingemans, Bruna & van Furth, 2002). The complications of binge eating – excessive weight and health problems related to excessive weight (e.g., morbid obesity; diabetes; pulmonary and cardiac problems) – are the usual reasons people seek treatment (Dingemans et al., 2002).

BED also negatively impacts quality of life in a variety of domains, including physical, emotional, social, and personal well-being (Tanofsky-Kraff et al., 2013). Individuals with BED may avoid social events, especially in situations where food is present, because of negative emotionality or out of fear they will binge in front of others (Smith & Robbins, 2013). Typically, binge eating is secretive, accompanied by a negative mood and cognitive rigidity, and may be an attempt to cope with negative emotionality (Sim et al., 2010). Risk factors for BED may include the personality traits of neuroticism and urgency, impulsivity, a tendency toward experiencing negative emotionality and a socio-cultural environment that enforces the ability of food to reduce negative emotionality (Ferriter & Ray, 2011). Individuals with SUDs also score highly on measures of impulsivity, sensation seeking, and have high levels of emotional dysregulation (Grant, Potenza, Weinstein & Gorelick, 2010).

Research has cited high comorbidity rates between BED and SUD. About one-fourth (24.8%) of individuals with BED report having a lifetime SUD, while 2.7% have a current SUD. Heightened levels of SUDs have also been found in relatives of those with BED (Lilenfeld, Ringham, Kalarchian & Marcus, 2008). Greater lifetime and current SUD comorbidity rates have been cited in males with BED (40.4% and 5.3%, respectively) compared to females (20.0% and 1.9%, respectively) (for rates of the co-morbid disorders, see Grilo, White & Masheb, 2009), suggesting that gender may factor into the overlap between BED and SUD.

## DIAGNOSIS AND ASSESSMENT

For the first time within DSM history, BED is formally included as a disorder in the DSM-5. Previously BED was classified as a *disorder for further study* in Appendix B of the DSM-IV. The following are the diagnostic criteria for DSM-5 (American Psychiatric Association, 2013) binge eating disorder; A) Recurrent binge eating episodes which are experienced as having a loss of control over eating, occur within a discrete period of time (within 2 hours), and involve eating a significantly larger amount of food than most people would eat in a similar time period; B) Binge eating episodes that are associated with at least three of the following: eating rapidly; eating until uncomfortably full; consuming large quantities of food when not physically hungry; eating alone due to embarrassment; and/or feeling disgust, depressed, or guilty post-binge; C) Binge eating produces distress; D) Binge eating episodes occur at least one time per week for three months; and E) Binge eating is not accompanied by compensatory behavior. Severity of binge eating disorder is classified as the following: *mild* – 1 to 3 binge eating episodes per week; *moderate* – 4 to 7 binge eating episodes per week; *severe* – 8 to 13 binge eating episodes per week; *extreme* – 14 or more binge eating episodes per week.

The following are the diagnostic criteria for DSM-5 (American Psychiatric Association, 2013) substance-related and addictive disorders, which encompass 10 different classes of drugs. A) Using substances for a greater amount of time or using a greater quantity of substance than intended; B) Despite intent to decrease substance use, inability to reduce or regulate substance use; C) A large amount of time is occupied by obtaining, using, or recovering from the effects of the substance; D) A craving is experienced to use the substance; E) Work, school, or home obligations are interfered with due to substance use; F) Consistent social and interpersonal problems result from substance use; G) Withdraw from social, occupational, or recreational activities due to substance use; H) Continued use of substances despite the negative physical and psychological consequences; I) Increased substance tolerance over time; J) Experience withdrawal symptoms when ceasing substance use. *Mild* severity substance use is categorized as the presence of 2 to 3 symptoms, *moderate* is 4 to 5 symptoms, and *severe* is 6 or more symptoms.

Similarities noted between these diagnostic symptoms include eating larger amounts of food than intended; inability to decrease binge eating, despite concerted efforts; spending substantial amounts of time binging or recovering from the effects of binging; reducing other pleasurable activities due to binging; and binging despite persistent negative consequences (Cassin & von Ranson, 2007). Both BED and SUD are triggered by cravings/urges and affective states and may serve to regulate negative affect (Gearhardt, White & Potenza, 2011; Luce, Engler & Crowther, 2007). These diagnostic and behavioral phenotypic similarities support the similarities between BED and SUD, but do not necessarily mean that they should be categorized similarly.

When applying SUD diagnostic criteria to BED, several issues arise. First, the concept of “out of control” differs. Consuming a greater amount of alcohol than intended describes the “out of control” nature in SUD, which is somewhat subjective given the use of the word “intended”. In contrast, the “out of control” in BED refers to consuming an objectively large amount of food in a discrete time period (Gearhardt et al., 2011), which is more objective in nature due to the wording “discrete time period”. Furthermore, a large amount of food eaten throughout the day may not be diagnostically considered “out of control”, whereas consuming a large amount of alcohol throughout the day may be considered “out of control”.

SUD criteria also differentiate between 10 different substances, whereas BED diagnostic criteria do not specify the addictive component of food (American Psychiatric Association, 2013; Gearhardt et al., 2011). Differentiating between substance types is useful, since addictive substances have a different specific effect on the brain (i.e., opiates target opioid receptors). Since food is a mixture of macro- and micronutrients, food has a widespread impact on the body and impacts multiple brain receptors (see Volkow, Wang, Tomasi & Baler, 2013 for a review on how food differentially impacts the body). Potential candidates for addictive substances include sugar and other refined sweeteners, fat, refined carbohydrates, and salt (Ifland et al., 2009). The mechanisms of how each nutrient in food influence the neurobiological pathways of reward and motivation systems are not well understood due to the complex nature of metabolism. However, through animal models, researchers are beginning to understand how sugar and fat impacts various neurological pathways (see the *Neurobiological underpinnings* section below). Additionally in BED, food-seeking motivation and behaviors may come from individual factors, such as eating related cognitive distortions, body weight concerns, and dietary restriction, as well as excessive consumption of highly processed food (Gearhardt et al., 2011).

## FOOD ADDICTION AND BINGE EATING

Currently, clinicians and researchers are discussing the validity of characterizing BED as a food addiction (FA). Proponents of food addiction describe the similar neuro-biological underpinnings of BED and SUDs (see the *Neuro-biological underpinnings* section below for more details) and highlight the similar experiences between the two populations, such as an out of control feeling, urges, and triggering emotional states (Gearhardt et al., 2011).

The development of the Yale Food Addiction Scale (YFAS; Gearhardt, Corbin & Brownell, 2009) highlights the similarities between food addiction and overeating. The YFAS modified the DSM-IV substance dependence criteria for food (see Gearhardt et al., 2009 for these criteria) and classifies individuals as having FA by meeting three or more diagnostic criteria and at least one of two clinically significant items (for a more detailed review see Ziauddeen & Fletcher, 2012).

Using the YFAS scale, 56.8%–72% of individuals who met FA criteria, also met criteria for BED; while only 24% who did not meet FA criteria, met diagnostic threshold for BED (Davis et al., 2011; Gearhardt et al., 2011). Participants meeting both FA and BED criteria were found to have a greater propensity towards impulsivity and hedonic eating (Davis et al., 2011). Using the DSM-IV substance abuse criteria modified for binge eating, however, Cassin and von Ranson (2007), who found that a majority (92.4%) of individuals with BED met diagnostic standards for “food addiction”. While these results highlight similarities between FA and BED, they also suggest the possibility that FA exists in a subset of individuals with BED (possibly in the highly impulsive individuals with BED) and the need for well-established definition and criteria for FA in order to progress research.

## NEUROBIOLOGICAL UNDERPINNINGS OF BED AND SUD

Understanding the neurobiology underlying BED may help clinicians better understand the nature of the disease, as well as how to best classify this disorder. Both BED and SUD have been associated with reduced activity in the orbito-frontal and prefrontal cortex areas, which are associated with self-control, as well as dysfunctional dopamineric and opioid pathways (Smith & Robbins, 2013). Polymorphisms on the OPRMI mu-opioid receptor gene (A118G) and DRD2 dopamine receptor gene (Taq1A A1) have been found in both samples of BED and SUDs, and may increase their vulnerability to the reward characteristics of food and substances (Davis et al., 2009; Wang, Volkow, Thanos & Fowler, 2004).

## Dopamine and opiate hypotheses

Dopaminergic and opioid pathways both play an important role in feeding behavior and the maintenance of a SUD. Dopamine is involved in the motivation to participate in rewarding activities, as well as influencing conditioned reinforcement and prepotent conditioning in the mesolimbic dopamine system; opiates are involved in the enjoyment of the rewarding activity (Davis et al., 2009; Wise, 2004). These neurotransmitter pathways, however, do not function independently, but are rather intertwined. Dopaminergic signals can be altered by other neurotransmitters, such as endogenous opioids, and some opiate signal pathways need a functional dopamine D2 receptor to function (Davis et al., 2009).

Researchers have used animal models to better understand the role of dopaminergic and opioid pathways in the complex relationship between feeding and addictions. For example, sucrose-dependent rodents display delayed satiation, consume more sucrose, and release more dopamine in the accumbens shell (Davis & Carter, 2009) as well as blunted activity in the cortical-striatal regions (Hommer et al., 2012). Similarly, both drugs of abuse and highly palatable food (high in sugar and fat) can increase dopamine levels, enhancing the stimulus-reward association (Davis & Carter, 2009; Koob & Le Moal, 2001). Studies have found that both overfeeding and extended access to intravenous cocaine or heroin self-administration were related to the disruption of reward processing and diminished reward (Johnson & Kenny, 2010). Additionally, subsequent to 12 hours of intermittent food deprivation, rodents consume a high sucrose solution in a binge-like manner and ingest the majority of their daily caloric intake within the first hour of access, which parallels drug consumption behaviors of drug-dependent individuals (Avena, Rada & Hoebel, 2008). Furthermore, decreased consumption of and preference for, high fat/high sugar foods have been found after the administration of opioid receptor antagonists (Cottone, Sabino, Steardo & Zorrilla, 2008), while an increased preferential intake of high-fat/high sugar items after injections of opioid agonists in the striatum was found in satiated rodents (Zhang, Gosnell & Kelley, 1998). Overall, these studies suggest that both the consumption of food and drugs may be an attempt to alleviate the reduced reward (Johnson & Kenny, 2010) and that individuals may use drugs of abuse and highly palatable food as a means of self-medicating a hypoactive dopaminergic system.

Similar somatic withdrawal symptoms have been found in rodents who were raised in a sucrose binge model after removing sucrose access and after administering an opioid antagonist (naloxone) and after food is removed for 24 hours (Avena, Rada & Hoebel, 2009). Neurochemically, similar alterations in dopamine and opioids are found in withdrawal from sucrose binges and from morphine, nicotine, and alcohol (Avena et al., 2009). Fat binging, however, has not been found to result in the opiate-like withdrawal symptoms as seen with sucrose. The differential impact of fat binging could be a result of the activation of the inhibitory neuropeptide galanin-induced by fat binging, which may inhibit the opiate-like withdraw response (Avena et al., 2009). Thus in moving forward in examining the overlap between BED and SUD, differentiating between how the different types of macronutrients (i.e. sugar and fat) impact reward pathways and receptor types may be beneficial.

For example, research into the specific receptor subtypes (mu, delta, and kappa) of the opioid receptor system has particularly implicated the mu-opioid receptor, which is involved in appetite regulation and hedonic processing of food related stimuli (Chamberlain et al., 2012) in BED. Specifically, the antagonism of this receptor has led to short-term reductions in palatable/high calorific food (in individuals with binge eating) and clinical alcohol use, as well as attentional bias for food cues (Chamberlain et al., 2012). A further examination of the different opioid receptors may aid in understanding of the crossover between binge eating and addiction.

Pharmacotherapies targeting the mu opioid receptor have illustrated the potential benefits of opiate antagonists in the treatment of BED, in that studies have found that opiate antagonists decrease food intake and food seeking behavior in human and animal models (Cambridge et al., 2013). Gar-butt, West, Carey, Lohr and Crews (1999) hypothesized that naltrexone may work through decreasing the emotional response to alcohol consumption; thus it is possible that naltrexone may also alter one's subjective emotional response (i.e., decrease the pleasantness of palatable food) to food, which may lead to decreased binge episodes (Rabiner et al., 2011). Only one randomized, placebo-controlled trial included individuals with BED, which found decreased binge duration and frequency in obese binge eaters taking naltrexone (at dosages of 100–150 mg/d); however, the naltrexone group was not significantly differentiated from compared to placebo (Alger, Schwalberg, Bigaouette, Michalek & Howard, 1991). Yet, three case reports found naltrexone were useful in treating BED using higher dosages of 200 to 400 mg/day (Marrazzi, Markhan, Kinzie & Luby, 1995) and augmentation with fluoxetine (Meyer, 2008; Neumeister, Winkler & Wober-Bingol, 1999). A similar medication, naloxone, which is an opiate blocker and is proposed to decrease reward response, has been useful, however, in decreasing intake of high sugar and high fat food in both lean and obese binge eaters (Drewnowski, Krahn, Demitrack, Nairn & Gosnell, 1995).

One of the most widely studied pharmacotherapeutic intervention for SUDs are opioid antagonists (such as naltrexone) and partial agonists (such as nalmefene) which have been found to be effective in the treatment of patients with alcohol and nicotine dependence. Double-blind, randomized, controlled trials have found that compared to placebo, naltrexone may help reduce number of days drinking, amount of alcohol consumed, number of relapses (Snyder & Bowers, 2008), and especially in those who complete treatment. King, Cao, Zhang and O’Malley (2013) recently found that nicotine-dependent females treated with naltrexone had significantly less weight gain at a one-year follow-up after achieving abstinence from nicotine, suggesting that opioid antagonist treatment may decrease urges prompting binge eating episodes *after* cessation from substance addiction.

Minimal research has explored dopamine in the treatment of BED. Past research found bupropion, a selective reuptake inhibitor of dopamine and norepinephrine, effective for decreasing nicotine and food related cravings (Brody et al., 2004; Jain et al., 2002; Jorenby et al., 1999). Only one randomized, controlled trial has examined bupro-pion in BED, finding that the active medication did result in short-term weight loss compared placebo, but was not associated with decreases in binge eating or food cravings (White & Grilo, 2013). In a double-blind study of sustained-released bupropion for smoking cessation in a large sample, researchers found that bupropion was superior to placebo in decreasing smoking (Sheng et al., 2013).

Overall, a fair amount of research has investigated the role of the dopaminergic and opioid pathways in the motivation and reward of food and drug related behaviors through animal models; however, pharmacological human trials modifying these neurotransmitters are relatively novel for BED and need further investigation. Based on research presented above, targeting the opioid pathways show promise and future studies may want to further explore the use of opioid antagonists that alter the mu-opioid receptor in treating BED.

## Serotonin – Acute tryptophan depletion (ATD) hypothesis

Low levels of tryptophan have consistently been shown to result in carbohydrate/fat rich, highly palatable food binges (Corsica & Spring, 2008). Tryptophan, the most scarce amino acid, is essential for serotonin synthesis and is greatly increased after consuming a meal high in carbohydrates (through the action of insulin; Fortuna, 2012; Schaechter & Wurtman, 1990). Theories suggest that low baseline serotonin levels decrease inhibitory control over food and drug cravings and heighten the desire for sweet foods, opposing impulse regulation when exposed to rewarding (i.e., highly palatable) foods (Fortuna, 2012). Using animal models, Asin, Davis and Bednarz (1992) found decreased sucrose in-take with the administration of increasing dosages of serotonin agonists. Greater serotonin levels (as a result of D-fenfluramine) were also associated with the reduction of smoking withdrawal symptoms (such as increased carbohydrate intake and weight gain) in overweight females (Spring, Wurtman, Gleason & Kessler, 1991). Heightened alcohol cravings have been associated with lower levels of serotonin availability at synapses and with acute tryptophan depletion (Ait-Daoud et al., 2009).

Overall, selective serotonin reuptake inhibitors (SSRIs) have been found to decrease binge eating and weight symptoms, as well as improved mental health in individuals with BED in eight randomized, placebo-controlled trials that lasted between 6 and 20 weeks (McElroy, Guerdjikova, Mori & O’Melia, 2012). Researchers have found that in samples of individuals with DSM-IV BED, fluvoxamine and sertraline were related to significantly reduced binge frequency and BMI, as well as decreased severity of BED compared to placebo (Hudson et al., 1998; McElroy et al., 2000). Contrarily, little evidence, however, supports the use of SSRIs in treating individuals with alcoholism, with three of four randomized controlled monotherapy trials not showing any significant differences between active medication and placebo (Miller, Book & Stewart, 2011). SSRIs are also not recommended for either first or second-line treatments for nicotine, opioid, cocaine, or cannabis dependence (van den Brink, 2012). Since SSRIs may benefit some with BED, while not much evidence supports treating SUDs with SSRIs, this suggests that differing neurobiological mechanisms are dysfunctional in BED versus SUD and that BED may have more overlap with mood disorders than SUD does with mood disorders.

## Glutamate hypothesis

Glutamate has been found to play a role in the regulation of food intake, drug seeking behavior, and modifying binge eating (McElroy et al., 2011; Tata & Kockler, 2006). More specifically, increased food intake has been associated with the blockage of glutamate receptors within the nucleus accumbens (Maldonado-Irizarry, Swanson & Kelley, 1995). In animal models, restoring glutamate activity to normal levels, decreases cocaine- and heroin-seeking and slows reconditioning to alcohol and cocaine (Baker et al., 2003; Myers, Carlezon & Davis, 2011). Both acamprosate (a glutamate N-methyl-D-aspartate receptor antagonist) and topiramate (a glutamate kainate receptor antagonist) have been explored as treatment options for both BED and SUDs.

Acamprosate, which is a glutamate N-methyl-D-aspar-tate receptor antagonist, may reduce mGluR5 function, and is approved for alcohol dependence (Miller et al., 2011), has been found to decrease food craving and weight gain in individuals with alcoholism (McElroy et al., 2011, 2012). Acamprosate has been used in one randomized, placebo-controlled trial for BED, and fourteen trials have compared acamprosate to placebo in individuals with alcoholism. For the one BED trial with acamprosate, McElroy et al. (2011) found that although acamprosate was not associated with significantly decreased binge frequency, it was associated with secondary measures of binge day frequency, food craving, and obsessive–compulsive symptoms of binge eating. BMI was also significantly decreased in participants receiving the active medication compared to placebo (McElroy et al., 2011) suggesting acamprosate may benefit some individuals with BED. Even though acamprosate has been approved for alcohol dependence, overall, randomized, placebo-controlled trials have only provided mixed evidence regarding efficacy, with seven studies favoring acamprosate and seven providing mixed results or no significant differences when compared to placebo (Miller et al., 2011).

Topiramate is an antiepileptic drug found to be useful for alcohol dependence, weight-loss, and BED (Claudino et al., 2007; McElroy et al., 2003, 2007) and has been examined in three randomized, placebo-controlled trials. Two of the three trials found that compared to the placebo-group, those receiving topiramate had significant reductions in binging frequency (Claudino et al., 2007; McElroy et al., 2007). Even though one trial found that topiramate did not decrease binging frequency, all three trials found that the topiramate group had greater weight loss and a higher remission rate of binge eating compared to placebo (Claudino et al., 2007; McElroy et al., 2003, 2007).

As previously mentioned, topiramate has demonstrated promising efficacy for alcohol dependence as well. In a randomized, double-blind study of 150 individuals with alcohol dependence, researchers found that topiramate treatment resulted in significant improvements in all drinking measures, as well as an objective biochemical measure of alcohol, compared to placebo (McElroy et al., 2003). Further, a meta-analysis of 3 placebo-controlled trials of topiramate and alcohol dependence found that topiramate groups had significant improvement in subjective and objective measures of alcohol dependence, such as significantly lower rates of g-GT and significantly more days of alcohol abstinence and fewer days of high alcohol consumption (Arbaizar, Dierssen-Sotos, Gómez-Acebo & Llorca, 2010).

Targeting the glutamatergic system with treatment may provide a promising treatment for some individuals with BED, especially in those who are obese. Moving forward, researchers may want to further investigate glutamate based treatment, such as topiramate and acamprosate, for BED.

## NEUROBIOLOGICAL UNDERPINNINGS OF BED AND OBESITY

Approximately 36% of binge eaters have been obese in their lifetime (Gadalla & Piran, 2007), while between 23%–46% of obese individuals report BED (Bulik, Sullivan & Kendler, 2002), indicating that obesity and BED are not synonymous. Examining the neurobiological similarities and differences between BED and obesity may help researchers understand how BED diverges from SUDs. Similar to SUDs, recent research using functional magnetic resonance imaging suggests a subgroup of obese individuals with binge eating disorder exists that have hypoactivity in the ventromedial prefrontal cortex, inferior frontal gyrus, and insula; areas of the brain highly implicated in reward and motivational behaviors and associated with impulse control (Balodis et al., 2013).

## Dopamine

As previously mentioned BED has been associated with altered dopaminergic functioning. Dysfunctional dopamine may also be associated with obesity. In animal models, one study found that rats (both obesity-prone and obesity-resistant rats) increased their intake of high fat food, while, decreasing their intake of “regular” food after receiving bromocriptine (a dopamine, DRD2, agonist). At a dosage of 20 mg/kg, both obesity-resistant and obesity-prone rats pressed the food level during the non-reinforcement period at significantly greater rates compared to their baseline; whereas only obesity-prone rats increased these lever presses at bromocriptine dosages of 10 mg/kg of body weight. These results underscore dopamine’s role (specifically the D2 receptor) in increased motivational drive for highly palatable food and suggest that treatments targeting DRD2 activity may be useful for impulsive, obesity-prone individuals (Thanos, Bermeo, Wang & Volkow, 2011).

In human trials, some studies have found a negative association between BMI and striatal D2R binding and a positive correlation between striatal D2R binding and prefrontal metabolism, suggesting that both a dysfunction in the reward pathway and reduced inhibitory control may contribute to obesity (Volkow et al., 2008; Wang et al., 2001). However, the negative association between BMI and striatal dopamine binding were not replicated in a more recent study when comparing overweight or obese individuals to normal weighted controls (Haltia et al., 2007). One possible explanation for these differences could be that there was a large difference between sample BMIs, with the initial study having a mean BMI of 51.2 and in the more recent study, over-weight males and females had a mean BMI of 31.8 and 34.4, respectively. Yet in 2011, Wang et al. found that baseline levels of the dopamine D2 receptor availability in the striatum did not differ between binge and non-binge eaters and had no correlation with BMI. Thus, studies comparing individuals with and without BED provide heterogeneous neurobiological results in regards to dopamine and cannot specifically be applied to the distinctiveness of BED. See Ziauddeen, Farooqi and Fletcher (2012) for a comprehensive review of this data.

Treatments targeting dopamine in treating obesity are also relatively recent and few trials have been completed. Bupropion, a selective reuptake inhibitor of dopamine and norepinephrine, has been found effective (at dosages of 300 mg/d and above) in placebo-controlled trials which involved overweight and obese subjects taking the bupropion, coupled with a 400 to 600 daily calorie deficit, reporting significant weight loss when compared to placebo (Anderson et al., 2002; Gadde et al., 2001; Jain et al., 2002). The combination of bupriopion and naltrexone has also been found to significantly decrease weight over a 48-week period in a sample of obese patients (Greenway et al., 2009).

Based on findings from animal models and as well as human studies, dysfunctional dopamine pathways most likely play a role in the development of obesity and BED. Targeting dopamine receptors when treating BED may be useful, however, further research needs to be completed to better understand the nature of the specific dopaminergic receptors that influence the development of BED.

## CONCLUSIONS

A level of overlap exists in the phenomenology, clinical characteristics, and treatment efficacy between substance dependence and binge eating disorder. Research into animal models has illustrated that dopaminergic and opioid pathways play similar roles in the hedonic and rewarding properties of drugs and food and that serotonin and glutamate may also be important in the regulation of food intake and substance use. With such a limited caché of *in vivo* or *in vitro* research exploring the pathophysiology of BED, however, conclusions as to the extent of the overlap between BED and SUD and the appropriateness of labeling BED (or a subgroup of individuals within BED) as a “food addiction” remain unknown.

A number of the diagnostic characteristics seen in BED overlap with SUD, including binging in larger than intended amounts, continuing to binge despite negative consequences, and reducing other pleasurable activities due to binging. The hedonistic quality to binging followed by shame or remorse may suggest similar neurobiological underpinnings to addiction in individuals with BED. Further, understanding whether age, gender, racial or ethnic background have an impact on the development of illness is important and largely lacking from the nascent BED literature. In regard to BED and addiction, further examining family history, comorbidity, and clinical characteristics between and within these disorders will aid in triaging patients for proper care, lessening the mental and physical health burden associated with prolonged binge eating and addiction.
